# Complex Formation in Three-Body Reactions of Cl^–^ with H_2_

**DOI:** 10.1021/acs.jpca.1c05458

**Published:** 2021-09-28

**Authors:** Robert Wild, Markus Nötzold, Christine Lochmann, Roland Wester

**Affiliations:** Institut für Ionenphysik und Angewandte Physik, Universität Innsbruck, Technikerstraße 25, 6020 Innsbruck, Austria

## Abstract

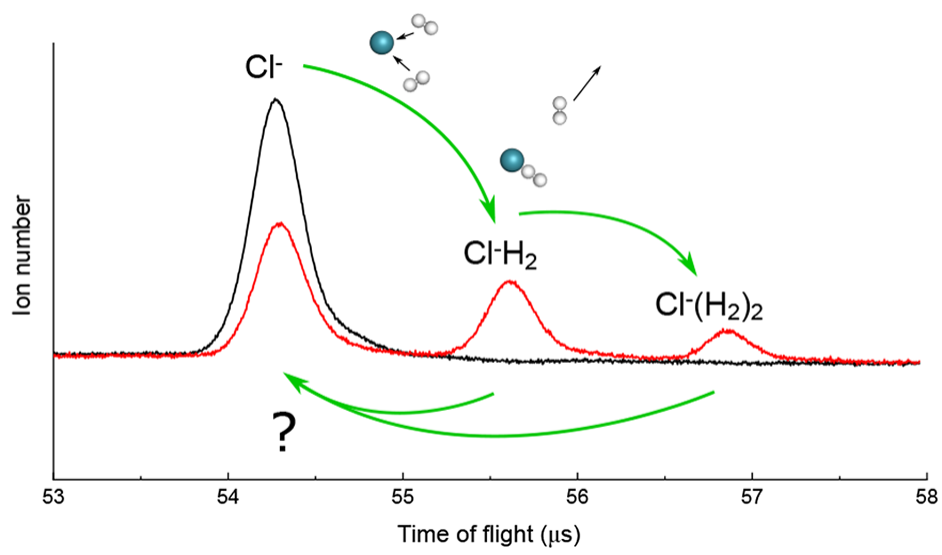

Three-body reaction rates of Cl^–^ with H_2_ to form the weakly bound complex Cl^–^(H_2_) are measured between 10 and 26 K in a linear radio-frequency wire trap. Formation of larger clusters of the form Cl^–^(H_2_)_2_ are also observed. The three-body (or termolecular) rate coefficients follow the form *aT*^–1^, with *a* = 1.12(2) × 10^–29^ cm^6^ K s^–1^. Reverse reactions to repopulate the Cl^–^ parent ion were measured, even though the binding energy of the complex makes bimolecular dissociative collisions energetically inaccessible at low temperatures. The back-reaction was found to be proportional to the cube of the hydrogen density, suggesting that the dissociation mechanism depends on multiple collisions. Comparisons of the rate coefficients measured in a 16-pole wire trap and a 22-pole trap demonstrate significantly lower ion temperatures in the wire trap.

## Introduction

Weakly bound negative ion–molecule complexes have been studied for many years, as their photoelectron spectra provide detailed information about the interaction potential of the corresponding neutrals.^1,2^ In particular, the dihydrogen halide anion complexes F^–^(H_2_) and Cl^–^(H_2_) have been of interest, as they allow for the investigation of the transition state region of the F + H_2_ and Cl + H_2_ reactions.^3−6^ These complexes have also been studied by using vibrational predissociation spectroscopy,^7−9^ specifically the Cl^–^(H_2_) anion,^10,11^ and by using ab initio calculations.^12,13^

Rate coefficients for three-body ion–molecule reactions and their temperature dependence give important insights into the nature of ion–neutral collision complexes. Such rate coefficients are challenging to calculate quantum mechanically^14^ and difficult to model accurately by quasiclassical methods.^15^ To describe three-body recombination, the energy transfer model is often used. It proceeds via an intermediate collision complex of two reactants, which is stabilized by collision with a third body that removes the excess energy. The three-body rate coefficient is then determined in large part by the lifetime of the intermediate complex, which in turn depends on the degrees of freedom available to partition the excess energy.^16^ In systems with few degrees of freedom, the resulting low reaction rates can be difficult to measure due to the long interaction times and/or high reactant densities required. Ion traps are well suited for these measurements as trapping lifetimes on the scale of hours can be achieved and reactant densities are easily controlled.

In this work we report on measurements of the three-body or termolecular rate coefficients of the reaction
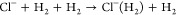
1in the range 10–30 K. Furthermore, the density and temperature dependence of the dissociation reaction of the complex back to the parent ion is presented, and possible mechanisms are discussed. We performed these measurements in two different ion traps, and different temperature effects of the two configurations on the ions are examined.

## Experimental Section

The experiments were performed in two different multipole radio-frequency (RF) traps mounted in the same experimental apparatus. A common 22-pole trap configuration was used initially and is described elsewhere.^17−19^ We have seen anomalous heating effects in this trap which we partly attribute to patch potentials from the trap rods.^19^ To minimize this effect and to improve optical access and increase experimental versatility, we constructed a 16-pole trap with 100 μm diameter wires as the RF trapping electrodes based on previous developments in our group,^20−22^ henceforth termed the wire trap. The wire trap replaced the 22-pole trap in the experimental apparatus, and reaction rate experiments were repeated. Results suggest that the ions reach a lower temperature in the wire trap, as described in the next section.

We create Cl^–^ in a plasma discharge of methyl chloride seeded in argon. A Wiley–McLaren spectrometer accelerates the ions toward the trapping region, and we selectively load ^35^Cl^–^ into the respective multipole trap by time-of-flight (ToF) mass separation. During loading the trap is at a potential of −250 V (wire trap) or −500 V (22-pole trap) to compensate for the energy gained by the ToF acceleration. Within the trap the ions collide with helium buffer gas that has thermalized with the trap’s copper housing, mounted on a closed cycle helium cryostat. Integrated heaters allow us to vary the trap temperature. We performed experiments at a minimum of 10 K to avoid freezing of the reaction partner H_2_ to the trap walls. Because the wires in the wire trap do not electrically shield the trapping region from the grounded trap housing as effectively as the rods in the 22-pole trap, the potential landscape in the wire trap at high voltage contains deep potential minima with quadrupole character that cause significant heating. To overcome this, we ramp the wire trap offset down to ground after loading, which corrects the trapping potential and significantly decreases the final ion temperature.

Once the ions are trapped and cooled, we add prethermalized hydrogen gas into the trapping region for varying amounts of time. We turn off the MCP detector during the H_2_ flow as described in Endres et al. to avoid arcing and damaging of the detector.^23^ Pressures of H_2_ are measured with a capacitive gauge connected to the trap housing via a Teflon tube. The capacitive gauge allows for an absolute pressure measurement independent of gas species. When calculating the gas densities in the trap, corrections for the temperature difference between the trap and the gauge, as well as thermal transpiration effects, were performed.^23^ The effects of thermal transpiration in the pressure regime in which we operate are empirically determined pressure corrections up to 30%. On the basis of these we estimate a 10% error in absolute density.

For unloading, the trap is returned to the same offset potential as during loading, which accelerates the ions out of the trap and allows for time-of-flight mass resolution of the product ions. We pulse a small gate voltage to deflectors situated close to the trap opening to allow only a small fraction of the ion cloud to pass undisturbed, producing a spatially localized ion packet. In the 22-pole trap setup we pulsed deflector plates, whereas for the wire trap we installed a wire gate made of 30 μm steel wires spaced 400 μm apart, with alternating polarities. This significantly increases the mass resolution.

## Results and Discussion

**Figure 1:**
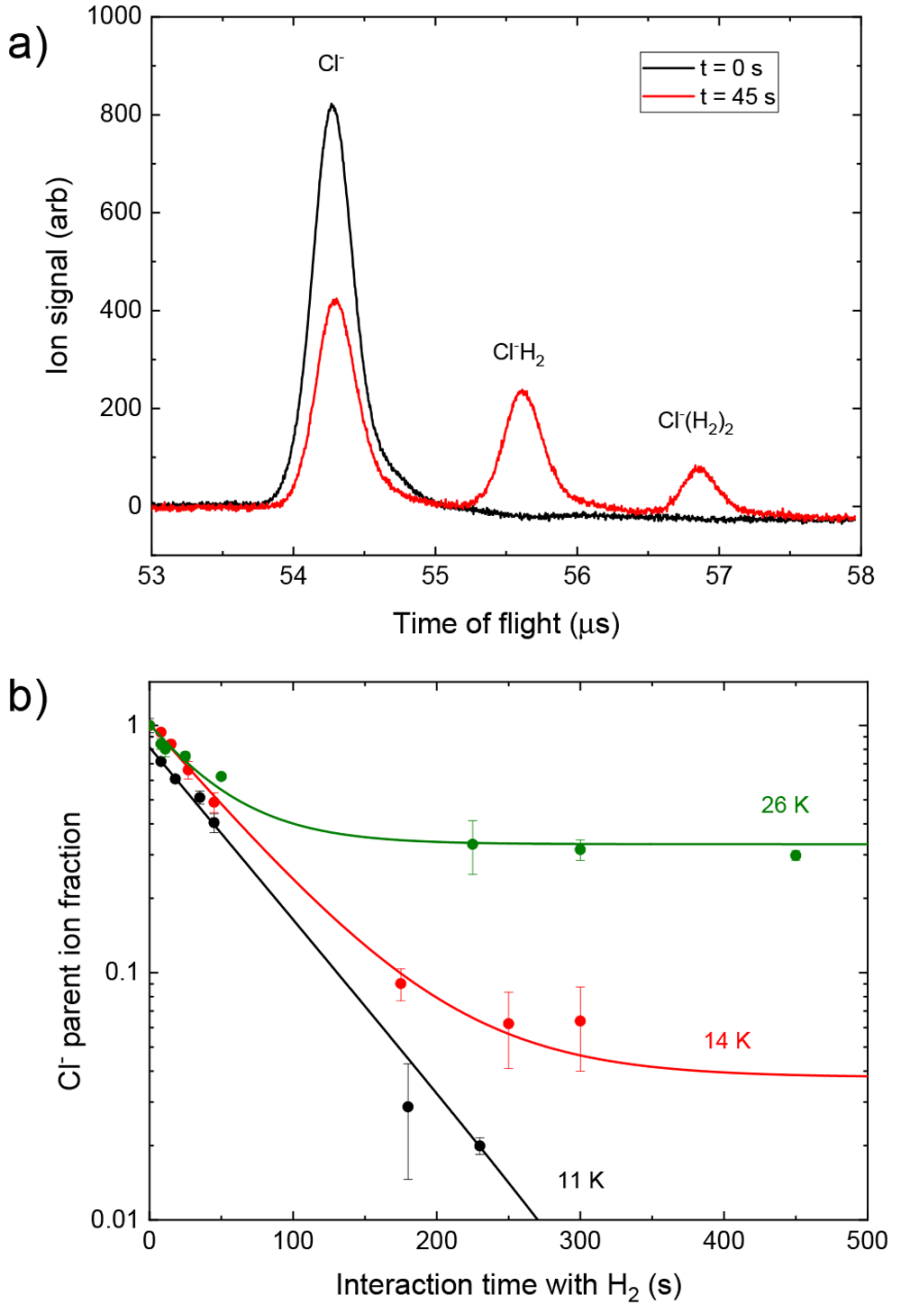
(a) An example of time-of-flight ion signals measured on the MCP detector, taken at 10 K at two different interaction times. Initially only the parent anion is present, which after interaction with H_2_ reacts to form higher clusters of Cl^–^(H_2_)_*X*_. (b) Examples of ion decay measurements at three different temperatures and similar densities, with fits to eq 3. The different steady-state values at long times give the ratio of the reaction rates *k*_bk_/(*k*_fr_ + *k*_bk_).

To measure the reaction kinetics of the trapped Cl^–^ anions, time-of-flight mass spectra of the trapped ions are recorded following different storage times in the trap. Two representative mass spectra are shown in Figure 1a for two different interaction times of the ions with H_2_ gas, which reveal well-resolved peaks of the parent ion and two hydrogen cluster products. When varying the interaction time between Cl^–^ and H_2_, we see a decay of the parent ion number (see also Figure 1b) and an increase of product cluster ions. Product clusters larger than Cl^–^(H_2_)_2_ are not measured.

For the determination of three-body reaction rates, we analyze the time-dependent decay of the parent ion Cl^–^. This decay is fitted to the following kinetics model with one forward and one backward reaction:
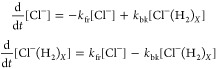
2Its normalized solution is
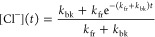
3where the forward reaction rate is *k*_fr_ and the backward reaction rate is *k*_bk_. In this step we ignore conversion between different-sized clusters and merely consider Cl^–^(H_2_)_*X*_ to be a reservoir from which Cl^–^ can be repopulated. We then vary the H_2_ densities and the trap temperature and repeat the decay measurements. Examples of data with fits to eq 3 can be seen in Figure 1b.

The model described by eq 3 focuses on the three-body reaction given in eq 1, which is discussed in next subsection. The ion–molecule complex formed by this reaction can further form larger clusters by collisions with H_2_. Additionally, collision-induced reactions from Cl^–^(H_2_)_*X*_ to Cl^–^ are seen, as evidenced by the steady state of Cl^–^ concentrations at long interaction times. These effects are discussed further below.

### Cl^–^(H_2_) Three-Body Formation Rate

**Figure 2:**
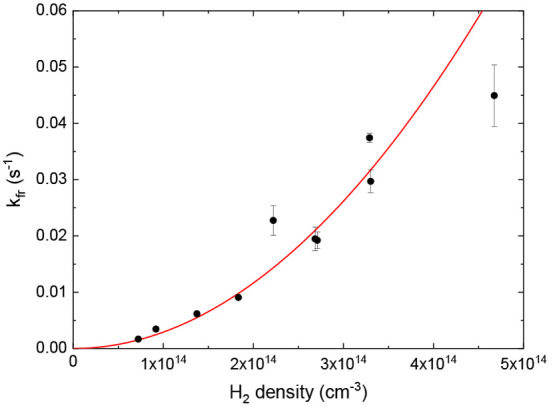
Dependence of the loss rates of the Cl^–^ ions as a function of H_2_ density in the 22-pole ion trap at 10 K. The solid line is a fit to a quadratic density dependence.

We have measured the three-body formation rate coefficient *k*_3_ for the anionic reaction system given in eq 1 at trap temperatures ranging from 10 to 26 K. An example of a three-body loss rate measurement as a function of H_2_ density, measured in the 22-pole trap, is shown in Figure 2. It exhibits the quadratic dependence on hydrogen density *n* expected from a three-body mechanism, *k*_fr_ = *k*_3_*n*^2^. Similar behavior was measured in the wire trap. Because of background Cl^–^ lifetimes on the order of hours, we do not measure an offset loss rate, and a linear component in the fit to the density dependence is consistent with zero. Hence, only a quadratic term is fitted to the loss rate data in further analysis.

**Figure 3:**
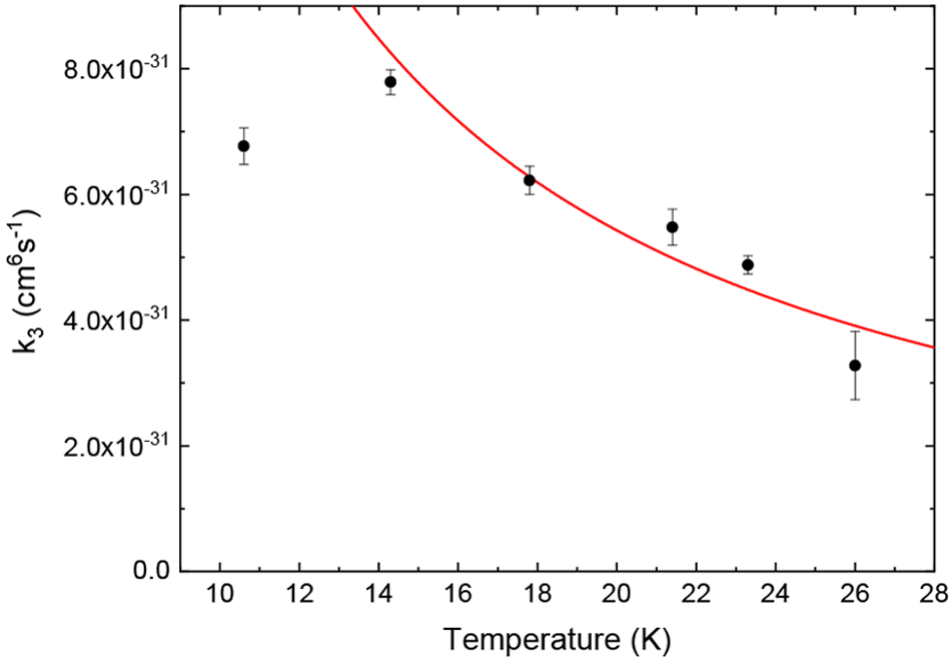
Measured three-body rate coefficients in the wire trap as a function of temperature. An inverse temperature dependence is found, with *k*_3_ ∝ *T*^–*n*^ and *n* = 0.98(9). The point at 10.6 K is not included in the fit, as discussed in the text.

The obtained temperature dependence of the fitted three-body reaction rate coefficients as measured in the wire trap is shown in Figure 3. We find three-body rate coefficients below 10^–30^ cm^6^/s for the measured temperature range. This is much smaller than previously measured three-body rate coefficients for OH^–^ with H_2_^24^ or Cl^–^ with CH_3_Cl,^25^ which can be attributed to the smaller density of internal states in the transient Cl^–^(H_2_) complex prior to stabilization. It also shows the sensitivity of ion–molecule kinetics measurements in cryogenic ion traps.

As a function of trap temperature we observe an increase of the reaction rate coefficient with decreasing temperature down to around 14 K. The lower rate constant at 10.6 K trap temperature is attributed to anomalous heating of the ions to an effective temperature around 16 K. Excluding this lowest temperature point, we fit the three-body rate coefficients to a power law *aT*^–*n*^ with *n* = 0.98(9). Fixing the exponent to *n* = 1, the resulting prefactor amounts to *a* = 1.12(2) × 10^–29^ cm^6^ K s^–1^. The exponent can be compared to theoretical models based on statistical treatments,^26−29^ on classical trajectories,^30^ or on full quantum scattering calculations.^31^ The latter two approaches, however, are not available for the present system involving an atomic ion and two diatomic collision partners. For an atomic ion with two atomic collision partners and neglecting internal excitations, a temperature dependence with *n* = 3/4 was found.^30^ Statistical models for one linear reactant predict a temperature dependence with *n* between 1 and 2, depending on how the contributions of ortho- and para-H_2_ are included.^26−28,32^ The measured value of *n* close to one may suggest that the hydrogen collision partners interact more atom-like than diatom-like, possibly because para-H_2_, which is described by a spherically symmetric rotational wave function, is more important for the three-body collision than ortho-H_2_. However, as the statistical models do not treat the rovibrational quantum-state-dependent collision dynamics properly, which should play an important role at the given temperatures, a more accurate theoretical treatment is required for a more detailed comparison.

In the 22-pole trap, three-body reaction rate coefficients have been measured that yielded a value of about 3.0(5) × 10^–31^cm^6^ s^–1^, independent of the trap temperature between 10 and 26 K. This value is smaller than those measured in the wire trap, which is a clear signature that the effective collision temperature in this trap was higher than in the wire trap. Experiments in multipole traps at cryogenic temperatures regularly find larger ion temperatures than predicted by simulation.^33^ Previous measurements of OH^–^ temperatures in the 22-pole trap exhibited a lower limit of about 25 K for both rotational^19^ and translational temperatures.^34^ The present three-body rate coefficient fits to the expected value at such an effective temperature and clearly shows that the collision temperatures reached in the wire trap are almost a factor of 2 lower.

Heating effects in multipole ion traps are qualitatively attributed to radio-frequency (RF) heating, i.e., elastic ion–neutral collisions in the presence of the RF-driven ion micromotion, and instability heating caused by the nonlinear dependence of the electric field on position.^18^ A quantitative understanding of the temperature increase could not be reached, yet. However, patch potentials on the trapping electrodes or the surrounding trap components might provide an explanation. The lower temperatures reached in the wire trap, where much less electrode surface is located in the vicinity of the ions, supports this notion.

Accurate theoretical calculations of the three-body rate coefficient for the formation of Cl^–^(H_2_) are not yet available. Instead, the measured three-body rate coefficient can be compared to a simple model calculation going back to Thomson.^35^ Here, a critical radius *b* between reactants is defined inside of which a collision with a third body can remove enough energy to stabilize the complex. This critical radius defines a collision time scale which provides the estimate for the three-body reaction without the formation of an intermediate complex, *k*_3_ ∼ *v*σ*b*, where *v* is the relative velocity and σ the estimated collision cross section.^35,36^ Taking the critical radius from the interaction potential gives a three-body rate coefficient ∼1 order of magnitude lower than measured values with a negligible temperature dependence. This suggests that the reactant ion and the first H_2_ molecule form a short-lived transient ion–molecule complex, which is subsequently stabilized by the second hydrogen molecule.

**Figure 4:**
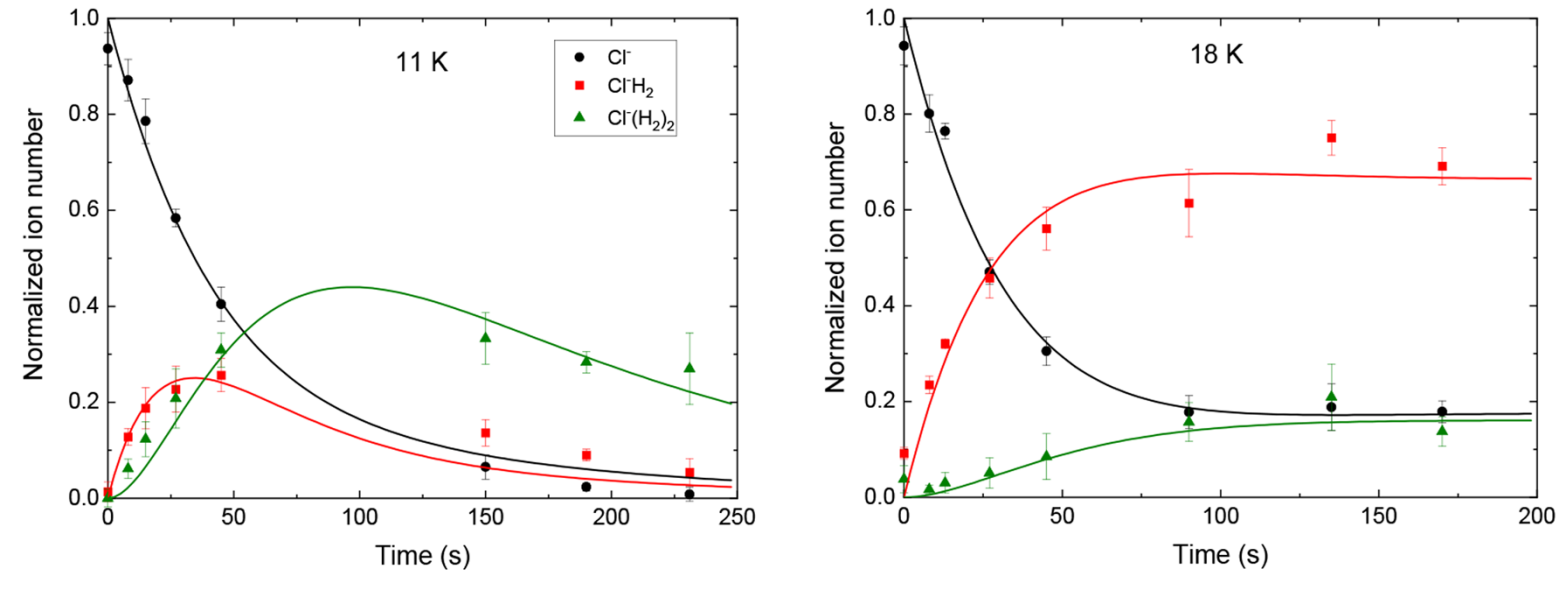
Normalized ion number as a function of time at two different temperatures. The fits to the three visible masses explicitly include individual growth and loss rates as well as growth to a reservoir of larger clusters. The left panel at 11 K shows loss to larger clusters, whereas in the right panel at 18 K the three visible peaks account for essentially all ions.

The model of eq 3 can be expanded to explicitly include reactions from Cl^–^(H_2_) to Cl^–^(H_2_)_2_ and from there to a reservoir of larger clusters as well as backward reactions to smaller clusters from each of these complexes. A numerical solution to the coupled equations can be fit to the three ion peaks of the recorded mass spectra, some examples of which are shown in Figure 4. At 11 K, the overall ion loss signifies formation of larger clusters outside of the recorded mass spectrum. At 18 K essentially all ions contain two or less H_2_ molecules, and above 18 K only Cl^–^ and Cl^–^(H_2_) are visible.

### Cl^–^(H_2_) Collision-Induced Fragmentation

**Figure 5:**
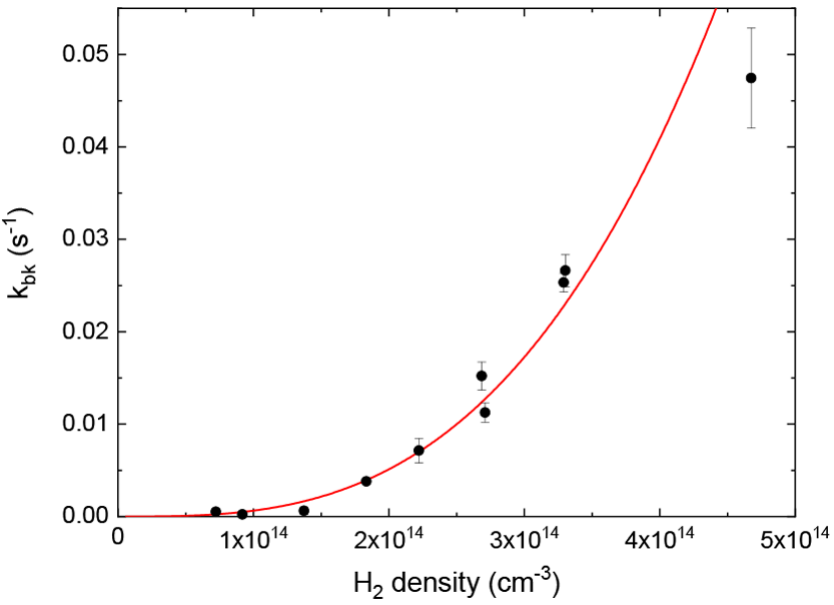
Fitted back-reaction rates *k*_bk_ as a function of H_2_ density in the 22-pole trap at 10 K trap temperature. The fit shows a clear *n*^3^ dependence, with constant, linear, and quadratic terms consistent with zero.

While the forward reaction is well-described by a three-body formation reaction, an unexpected density dependence was found for the back-reaction in eq 2. The values of *k*_bk_ from the data shown in Figure 2 are plotted as a function of H_2_ density in Figure 5. A power law fit revealed an *n*^3^ density dependence, and a polynomial fit to third order shows that constant, linear, and quadratic contributions to the back-reaction rates are zero to within the 1σ error.

To clarify the mechanism for the backward reaction, we have estimated the expected rate for collision-induced dissociation of Cl^–^(H_2_) in a single collision. For this we assume a Langevin capture rate multiplied with a Boltzmann factor to account for the endothermicity. The dissociation energy of the Cl^–^(H_2_) complex is calculated to be 52 meV for the Cl^–^ + para-H_2_ asymptote and 65 meV for the respective ortho-H_2_ asymptote.^37^ For dissociation to the para-H_2_ asymptote, single collisions at 10 K then produce a dissociation rate that is about 16 orders of magnitude lower than our measured values. Single collisions are therefore only expected to become significant above 30 K. This suggests that a different process causes the rapid destruction of the complex at low trap temperatures.

The measured *n*^3^ dependence of the Cl^–^(H_2_) fragmentation signifies that multiple collisions are necessary to remove the H_2_ and repopulate bare Cl^–^. Additionally, the collisions have to overcome the considerable binding energy of at least 52 meV for Cl^–^(para-H_2_). This suggests that the complex can be collisionally excited to higher-lying bound rovibrational states, in which the excitation can be stored until a subsequent collision can excite the complex further. The implication is that the competing quenching collisions would have to occur with a low enough probability.

Besides thermal energy, an additional energy source is rotationally excited ortho-H_2_ molecules, which represent 75% of the hydrogen molecules in the reactant gas. The energy difference between the lowest states of ortho- and para-H_2_ is 15.1 meV. If a collisional mechanism exists by which this rotational excitation is transferred to rovibrational excitation of Cl^–^(H_2_), such conversions could contribute a significant amount of energy to the dissociation. Previous data have suggested that such a mechanism is operative in OH^–^ collision with H_2_,^19^ albeit through an H^–^(H_2_O) intermediate complex.

**Figure 6:**
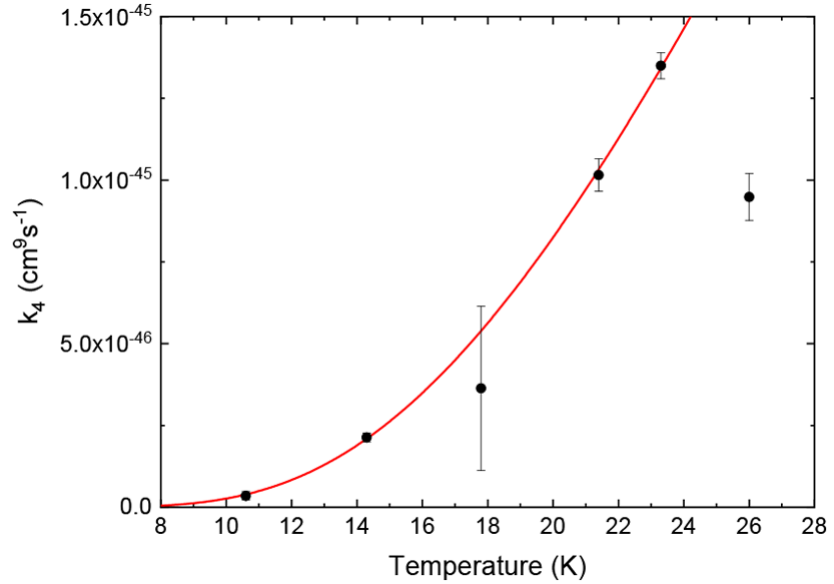
Strength of the *n*^3^-dependent back-reactions as a function of temperature. The fit, excluding the point at 26 K, is proportional to a Boltzmann factor with a binding energy of 5.9(1) meV.

If we assume an excited complex that is dissociated via a final bimolecular collision, then one would expect a behavior of the rate coefficient given by *k*_bk_ = *k*_4_*n*^3^ to follow the form *k*_4_ = *A*e^–*E*_b_/*kT*^, with a constant *A* and an effective binding energy *E*_b_ of the complex after internal excitation. The result of fitting *k*_bk_ for the different densities to a *n*^3^ dependence and plotting *k*_4_ as a function of temperature is shown in Figure 6. The data show good agreement to the fit to a Boltzmann factor with *A* = 2.6(2) × 10^–44^ cm^9^ s^–1^ and *E*_b_ = 5.9(1) meV, when excluding the data point at 26 K from the fit. At 26 K the temperature is large enough that fewer collisions are sufficient to dissociate the complex.

It is worth noting that the point at 10.6 K perfectly follows the temperature trend for the back-reaction, while it did not do so for the forward reaction. This suggests that the two processes are sensitive to temperature in different ways.

## Conclusion

Collisions between chlorine anions and molecular hydrogen have been investigated in a radio-frequency multipole trap at temperatures between 10 and 26 K. The reaction of Cl^–^ with H_2_ to form the bound Cl^–^(H_2_) complex proceeds via a three-body collision. The rate of this three-body reaction was measured as a function of temperature. The small rate coefficients, below 10^–30^ cm^6^/s, reflect the low density of states in the transient ion–neutral complex. The temperature dependence of the rate coefficient, proportional to T^–1^, roughly agrees with early statistical theories and the theoretical result for ion–atom–atom collisions but requires more accurate theoretical descriptions for a detailed analysis. Comparison of the rate coefficients measured in two different traps shows that the recently installed wire trap reaches significantly lower collision temperatures than the previously used 22-pole ion trap.

The collision-induced dissociation of the anion complex Cl^–^(H_2_) to regain bare Cl^–^ has also been measured. We find dissociation rates that are much higher than those predicted by bimolecular collisions and dependent on the cube of the hydrogen density. We argue that the required dissociation energy seems to be building up in rovibrational excitations that are partially resistant to quenching collisions, but further studies are necessary to confirm this. The temperature dependence of the dissociation suggests that the collision temperature is the relevant parameter in the dissociation mechanism, but the suppression of the three-body rate below 14 K is not yet understood.

We hope that this work stimulates theoretical calculations of absolute three-body rate coefficients and state-dependent collision-induced dissociation rates to compare with experiment and to identify the role of the internal quantum states in the transient complex.
